# Efficiency of probiotic culture consortium application for disinfection of dairy farm premises and prevention of mastitis in cows

**DOI:** 10.5455/javar.2023.j668

**Published:** 2023-06-30

**Authors:** Aikumys Zhumakayeva, Aygerim Zhubatkanova, Zhenisgul Asauova, Mereke Tokayeva, Zhomart Kemeshov

**Affiliations:** Department of Veterinary Sanitary, Saken Seifullin Kazakh Agrotechnical University, Astana, Kazakhstan

**Keywords:** Probiotic, strain, consortium, disinfectant, colonies

## Abstract

**Objective:**

In this work, the effect of probiotics on the state of the microbial background of the livestock building, on the state of udder teats, and on the prevalence of latent mastitis was investigated. Long-term use of the consortium has bactericidal activity against all cultures studied, especially the causative agent of mastitis, *Staphylococcus aureus*, except *Proteus mirabilis*, *Proteus vulgaris*, and *Escherichia coli*.

**Materials and Methods:**

The washes from animal housings and milk samples were collected from the dairy farms “Astana-Onim” Joint Stock Company and “Rodina” Limited Liability Partnership (Kazakhstan). The cleaning solutions and probiotic agents were applied directly to the udder teats of cows before and after milking. Diagnosis of subclinical mastitis was performed using the Kenotest rapid mastitis test. Directly counting the number of somatic cells in the collected milk samples from each cow was performed on a somatic cell counter.

**Results:**

Pathogenic microorganisms, including *S. aureus* bacteria (50% of samples) and bacteria of the *E. coli* group, *Enterobacter aerogenes*, and *P. mirabilis* (36% of samples), were detected on the udder skin and milk wipes. Using a consortium of probiotic microorganisms positively affects the mammary gland more quickly than using mastitis prevention agents alone. Probiotic use for a month resulted in a significant improvement in udder teat condition, with 60.7% of teats showing normal physiological reaction to milking, a decrease in complicated hyperkeratosis, and an increase in uncomplicated mastitis. The studies showed that in the experimental group, there was a 1.5-fold reduction in the number of cows with clinically pronounced mastitis. The experimental group showed no significant changes in the number of animals with high somatic cell levels before and after the study, while the control group without probiotics had a significant increase in diseased animals after 1 month.

**Conclusion:**

The use of a probiotic consortium has shown promising results in reducing the incidence of mastitis and improving milk quality in cows.

## Introduction

The priority task facing the livestock industry of the Republic of Kazakhstan is to ensure a high level of milk productivity of cattle and produce milk of high sanitary and technological quality. *Bacillus* and *Lactobacillus* are able to acidify the environment, and are antagonists of a number of microorganisms such as *Salmonella*, *Proteus*, *Staphylococcus*, *Escherichia coli*, pseudomonads, aeromonads, streptococci, yeast fungi, etc., with the help of producers, and also synthesize amino acids and vitamins [1,2].

Among the measures to increase the production of livestock products, improve their quality, and reduce their cost of production, the development and introduction of advanced technology in animal housing are of great importance. It is necessary to create conditions for animals in which they can best demonstrate the potential of their productivity due to their genetics. In cases of violation of keeping conditions, veterinary and sanitary norms, exposure to technological stress, etc., their productivity and resistance to diseases are reduced. In animals, the metabolism is disturbed, and the digestibility and assimilability of nutrients from forage are reduced, which negatively affects the efficiency of animal husbandry [3,4].

Currently, the search for alternative ways to replace and reduce the use of disinfectants on livestock farms is intensifying. One of the feasible directions is probiotics. Probiotics refer to collections of bacteria that exist in either vegetative or spore form and possess distinct antagonistic properties against harmful microorganisms in the body. Probiotics aim to create a healthy and stable microflora as opposed to an environment of unnatural absolute sterility. Probiotics have a beneficial effect on both animal and human bodies [5,6].

The use of certain antibacterial and disinfecting preparations negatively affects the quality of products, as they accumulate in livestock products [7–9]. Antibiotic treatment of lactating cows with subclinical mastitis is mostly ineffective against udder infections. Antibiotics, sulfonamides, contained in the preparations, irritate mammary gland tissue, inhibit local resistance, and violate the natural biocenosis of the mammary gland, which leads to the development of dysbacteriosis and frequent recurrence of the pathological process. For a long time, the components of drugs (antibiotics and sulfonamides) are excreted with milk, which poses a threat to human health [10]. Whereas biological preparations based on probiotic cultures do not accumulate in livestock products and contribute only to increasing the yield of meat and milk.

Probiotic preparations are promising alternatives to the use of chemotherapeutic agents. The bacterial strains have clearly expressed antagonistic activity against a wide range of pathogenic and conditionally pathogenic microorganisms; they secrete biologically active substances (enzymes and vitamins) necessary for the microorganism. Probiotics have no harmful side effects, are non-toxic, and can be used without restrictions. The utilization of probiotics may serve as a natural and efficacious substitute for the treatment and prevention of mastitis [11,12].

According to Barkova et al.’s research [13,14], the analysis of the data obtained 1 month after the experiment showed that the use of probiotic products had a positive effect on the udder condition in cows. In the experimental group, there was a significant increase in the percentage of animals with healthy udders (30.3%), which is 2.9 times higher than in the control group. In addition, the presence of hidden mastitis in cows was registered at a significantly lower level—39.7% in the experimental group, while in the control group, it was 54.2%.

Despite a large number of studies in the field of the creation of various drugs designed to treat and prevent mastitis in cows, researchers mainly focus on the development of antibiotic-containing agents for the treatment of mastitis. Little attention is paid to prophylaxis, in particular to udder teat treatment after milking. Nowadays, there are not enough preparations for mastitis prevention, especially those with probiotic cultures of *Bacillus subtilis* and *Enterococcus faecium* strains as active ingredients [15,16].

Therefore, the study of the application of probiotic disinfectants on dairy farms and their effect on the sanitary and hygienic parameters of milk is very relevant. Statement of novelty: A new type of biological agent from probiotic cultures is to be used as a cleaning agent for livestock facilities, as well as for the prophylactic treatment and prevention of mammary gland diseases like mastitis in highly productive cows. The purpose of the work is to study the effectiveness of biological and chemical disinfectants in dairy farms, and their impact on the incidence of mastitis in cows through the veterinary and sanitary evaluation of milk.

## Materials and Methods

### Ethical approval

The techniques used in this research were authorized by the Ethics Commission of S. Seifullin Kazakh Agrotechnical University (Regulation No. PEPPS SMK 11010/46-2013).

### The experimental base of the study

Materials for the study were washes from the places of animal housing and samples of milk obtained from the animals that were treated with biological cleaning agents. For the purpose of monitoring microbial contamination, we selected the dairy farms Astana-Onim Joint Stock Company and Rodina Limited Liability Partnership (LLP). There are 500 head of cattle on the farm. Every year, according to veterinary reports, 25–30 cows are diagnosed with mastitis. The livestock complexes of “Astana-Onim” and “Rodina” are free from infectious and parasitic diseases. Cows are examined for tuberculosis, brucellosis, and leukosis every year. Four times a year, cows are examined for subclinical mastitis, and sick cows are treated.

### Treatment and cleaning process of the livestock housing

The agents were applied by massage movements to the teat apex area in the amount of 0.5 gm per teat twice a day, directly after milking the cows. After that, we did not treat it with disinfectant. The condition of the teats of the mammary gland was assessed immediately before application of the indicated means, after 7 days of application, and also weekly within 2–4 weeks after completion of the treatment, to determine the prolonged action.

The effectiveness of preventive measures based on the use of probiotic products was evaluated based on model enterprises. Udder teats were treated before milking by washing the teats and mammary glands with 0.2%–0.5% solutions of Animal House Cleaner by Chrisal (Belgium). After milking, a 4% solution of PIP cow teat cleaner (PIP CTC) was applied by dipping them in a glass for disinfection or spraying teats with a sprayer. The active basis of these substances is represented by a complex of microbial strains: *B. subtilis*, *Bacillus pumilus*, *Bacillus licheniformis*, and *Bacillus megaterium*, which are strictly saprophytic aerobic microorganisms. A number of probiotic agents were included in the comprehensive program: PIP plus water for adding to water and spraying feed (0.5%), PIP CTC for treatment of cow udders, and PIP animal housing stabilizer (PIP AHS) for spraying livestock housing.

PIP AHS was sprayed using a 5-l sprayer with a telescopic nozzle. The preparation was sprayed on structural elements as well as animal decking in the volume of 1 l of concentrated solution per 300 m^2^ area. During the first 7 days, treatment of the room was carried out daily, then once every 3 days until the end of the test.

### Diagnosis of subclinical mastitis

The results were recorded based on a clinical examination of the mammary gland and a determination of the udder teat condition using a diagnostic scale. The presence of inflammatory diseases of the mammary gland was also determined using the Kenotest rapid mastitis test or direct somatic cell counting.

Pre-milking, palpation, and visual inspection all served to study the udder. The diagnosis of subclinical mastitis was performed using the Kenotest rapid mastitis test. The udder was cleaned with the alcohol wipe before the secretion was taken. The first streams of milk containing a large number of somatic cells were removed from each teat and placed in a separate dish. Then, a little milk was poured from each quarter of the udder onto a clean, preliminary disinfected plate, and 2 ml of reagent was added. The reagent was mixed with the milk with gentle circular movements. After a few seconds, we interpret the result obtained according to the instructions.

### Counting the number of somatic cells

Directly counting the number of somatic cells in the collected milk samples from each cow was performed on a somatic cell counter. A total of 225 milk samples from cows with different udder and teat conditions were analyzed. The somatic cell count was also analyzed on an ECOMILK scan (Bulteh 2000 Ltd., Bulgaria).

### Technological process of probiotic preparation production

The next stage of the work was the method of co-culturing active lactobacilli strains.

We used the following isolated biocompatible cultures for the work:

– *Bacillus subtilis* C; *Bacillus coagulans *P and *Bacillus amyloliguefaciens* E;

– *Lactobacillus helvetens* and *Lactobacillus acidophilus*.

The antagonistic activity was studied using indicator test cultures: *Salmonella typhimurium*, *Serratia marcescens*, *Staphylococcus aureus*, *E. coli*, *Proteus mirabilis*, *Pseudomonas aeruginosa*, *Bacillus cereus,* and *Candida albicans*.

Under laboratory conditions, 10 ml of each strain was prepared at a cell concentration of 9.0 lg CFU/cm^3^. Then, the culture liquids were combined and diluted with water in a 1:10 ratio. Thus, a working solution of culture liquid ready for application was obtained. The culture solution was sprayed by coarse-dropped irrigation with a spraying device. After 3 days, control seeding was performed by washing off the surfaces of floors, walls, feeders, drinkers, and iron partitions from animal housing. The activity of consortia was also determined by their ability to grow on cattle manure, as well as by their ability to neutralize unpleasant odors of manure.

The technological process of probiotic preparation production includes the following stages: obtaining biomass; obtaining cell suspension by centrifugation; preparing a protective medium; and mixing the protective medium with bacterial mass. In addition, a working concentration of the preparation capable of inhibiting the growth of bacterial cells was developed.

### Preparation of biomass of microbial strains forming part of the consortium

The strains selected for consortia produce specific types of useful enzymes, such as lipase for fat breakdown, protease for protein breakdown, and amylase for starch breakdown. In addition, these cultures have high antimicrobial activity [17,18].

Then, two types of microbial consortiums were compiled on their basis:

The first consortium included three cultures: *B. amyloliguefaciens* E, *B. coagulans* P, and *L. helveticus* T. The second consortium was composed of two cultures, including those of *B. subtilis* C and *L. acidophilus* B. To make a preparative form, we used consortium no. 2, consisting of two prebiotically active strains. Strains included in the consortium were grown according to the cultivation parameters developed earlier by “Ekostandart” LLP. *Bacillus subtilis *C strains were cultured on universal medium (dry enrichment broth).

*Lactobacillus acidophilus B on selective medium Man, Rogosa, and Sharpe agar (MRS)-1*

The strains were inoculated at a concentration of 100,000,000 cells/cm^3^ and incubated at 30°C for 48 h with a pH value of 6.7. The maximum biomass accumulation was 10^8^–10^9^ CFU/cm^3^ under these cultivation parameters.

*Centrifugation*

Further studies were performed to concentrate the cultures by sedimentation in a centrifuge. The culture liquid was centrifuged at 4,000, 10,000, and 13,000 rpm for 30 min. Next, the obtained culture precipitates after centrifugation were combined, thoroughly mixed, and then diluted with preserving medium in a 1:1 ratio. The preserving medium was 10% sterile saline.

### Analysis of surface contamination

The functionality of the finished biopreparation to reduce the growth and development of microbial infestations and odors inside the premises was investigated under laboratory conditions in model experiments.

For this purpose, a solution of the preparation was diluted at 1:10 and 1:100. For the analysis of floor surface contamination, the fingerprint or contact method, which is widely used to determine the biological contamination of the smooth surface, was applied.

To make imprints on the floor surface, pieces of filter paper cut into circles 3–6 cm in diameter were placed in Petri dishes, sterilized, and filled with molten agar medium. After cooling, the Petri dish was turned over with the side soaked with a medium on the surface under study. Then the petri dish with the agar was turned over with tweezers. Then the agar surface was additionally treated with the prepared working solution and placed for incubation on MacConkey plate agar (MPA) and MRS.

Petri dishes not treated with floor imprinting preparation were used as a control. The prints were incubated at 37°C for 2–5 days. Bacterial colonization was assessed by the number of colonies grown on the surface of the agar medium. This method of taking fingerprints differs favorably from the washing method in its ability to directly detect contamination of environmental objects and in the absence of microbial loss in the examined objects.

### Statistical analysis

The experiments were conducted in triplicate, and the results were presented as the mean ± standard error of the mean of three independent observations. The statistical analysis was carried out using Statistica 12.0 software (STATISTICA, 2014; StatSoft Inc., Tulsa, OK), with the one-way analysis of variance method used to determine differences between samples. The Tukey honestly significant difference test was utilized to compare means, and statistical significance was determined with a *p*-value of less than 0.05.

## Results and Discussion

The effect of treatment with probiotic cleaning agents is achieved through the colonization of treated surfaces with cultures of probiotic bacteria (*B. subtilis*), which inhibit the development of pathogenic microflora [19,20]. In the process of developing a consortium of probiotic cultures for use in the sanitation of dairy farms, we separated isolates from the skin flush of the udder of healthy cows. Using dilution, samples were seeded on nutrient media (selective plate agar, MPA, and MRS agar). Then, to obtain a pure culture, the samples were isolated by the starch method.

We selected 10 isolates of similar lactic acid bacteria and probiotic strains of bacilli from a set of colonies plated on various nutrient media for genotyping to create a consortium.

Microscopy was used to determine the species identity of the isolates. For microscopy, isolates were stained using the Gram technique. Thus, out of 10 cultures, 4 strains were identified as Gram-positive, chain-forming, spore-forming, and rounded-end bacilli. Six strains were Gram-positive, arranged singly and in a chain, and elongated with stumpy ends.

**Figure 1.**
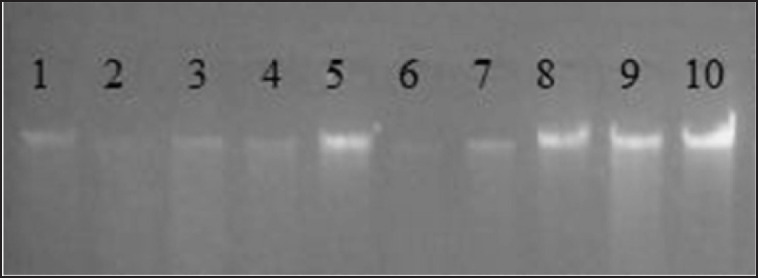
Gel electrophoresis of isolated DNA in agarose gel.

The species identity of the obtained pure colonies was determined by genotyping using 16S rRNA. For this purpose, deoxyribonucleic acid (DNA) was isolated from 10 isolates using the Power Soil DNA Isolation Kit (MO BIO). Then, we performed gel electrophoresis of the isolated DNA in a 1% agarose gel, according to Figure 1.

**Figure 2.**
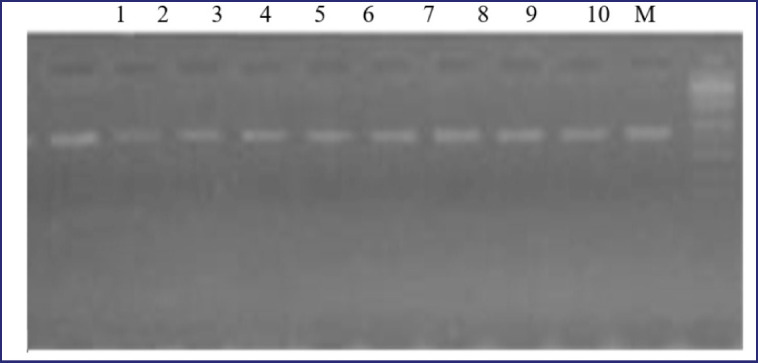
Gel electrophoresis of amplicons in 2% agarose gel.

The nucleotide sequence of 16s of the studied cultures was determined by polymerase chain reaction (PCR) amplification. PCR program: 92°C for 5 min, 92°C for 1 min, 52°C for 1 min 30 sec. 25 cycles, 72°C for 2 min, 72°C for 5 min, 15°C- ∞. The obtained PCR amplification results were fixed in a 2% agarose gel, according to Figure 2. Next, we sequenced the 16s gene on an Applied Biosystems genetic analyzer. The obtained nucleotide sequences were compared with the sequences of typical microbial strains in the Basic Local Alignment Search Tool Internet system. Data analysis showed a high level of homology (over 97%).

The antagonistic activity was evaluated by the size of the growth inhibition zone of the sensitive indicator culture around the colony of the tested strains of lactic acid bacteria and bacilli (Table 1).

**Figure 3.**
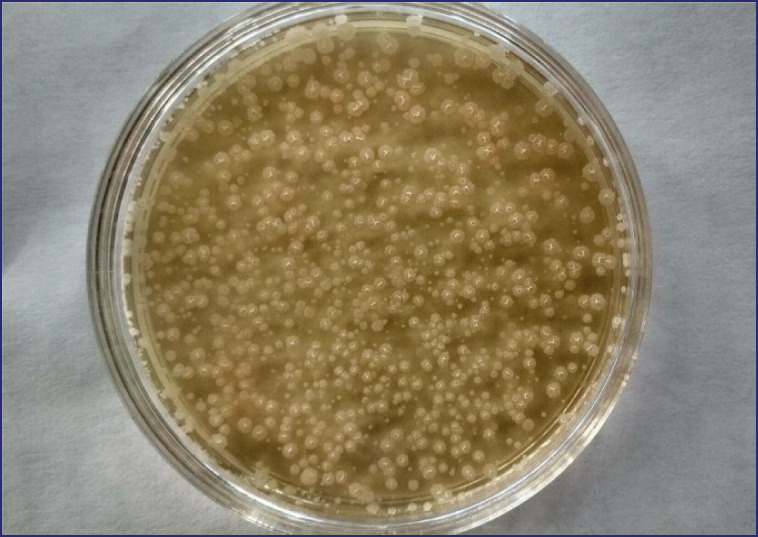
Plating of the probiotic culture *B. subtilis.*

The *B. subtilis* C strain was cultured on universal medium selective plate agar, and the *L. acidophilus* B strain was on selective medium MRS-1. The strains were inoculated at a concentration of 100,000,000 kl/cm^3^ and incubated at a cultivation temperature of 30°C for 48 h with a pH value of 6.7 (Fig. 3). Following the above cultivation parameters, the maximum biomass accumulation was 108 × 10^9^ COU/cm^3^.

Further studies were aimed at concentrating the cells by centrifugation and precipitation. The culture liquid was centrifuged at 4,000, 10,000, and 13,000 rpm for 30 min. The results of bacterial cell concentration before and after centrifugation are shown in Table 2. Cell precipitation at different rpm had a generally insignificant effect on cell titer. Centrifugation of strain *B. subtilis* C in the range from 4,000 to 13,000 rpm affected only the quantitative content of cells, while the degree remained at the same level. However, centrifugation of *L. acidophilus* strain biomass at high rpm had a negative effect on cell titer. Thus, at 13,000 rpm, a reduction in cell concentration was observed, despite the fact that lactic acid microorganisms are Gram-positive bacteria with a thick layer of peptidoglycans, which allows them to tolerate high centrifugal revolutions. At the same time, the level of cell concentration between 4,000 and 10,000 rpm was actually maintained at the same level. Based on the data obtained, it is recommended to precipitate the cells at 4,000 rpm for 30 min.

Then, the obtained culture precipitates after centrifugation were combined, thoroughly mixed, and then diluted with a preservative medium in a 1:1 ratio. The sterile 10% saline was used as a preservative medium. As a result, a concentrated form of biopreparation was obtained.

The main task of the technology of producing bacterial preparations based on living microorganisms is to provide such conditions for obtaining and processing the microbial mass that the maximum number of viable cells would be preserved in the finished product and their useful properties would not be lost [21,22].

For this purpose, the laboratory sample of concentrated biopreparation was stored at −20°C and +4°C. Currently, studies are underway to determine the assessment of cultural persistence and viability. The total microbial cell concentration of the biopreparation determined by the Koch method was 10^9 ^CFU/cm^3^. Control microscopy of the cells shows the presence of Gram-positive stripe-shaped cell forms in the smear preparation.

When evaluating the ability of the finished biopreparation to inhibit the growth and development of microbial infestations and odors inside the premises, the studies were carried out under laboratory conditions in model experiments. The working solution of the preparation was diluted 1:10 and 1:100, and the fingerprint or contact method was used. The pieces of filter paper cut in the form of circles 3–6 cm in diameter were imprinted on the floor surface and placed in Petri dishes with agar. Then, the agar surface was additionally treated with the prepared working solution and incubated on MPA and MRS. As a control, one Petri dish was left untreated with the preparation. The prints were incubated at 37°C for 2–5 days.

**Table 1.** Antimicrobial activity of probiotic strains.

Strains	Growth inhibition zones of test strains, mm							
	*E. coli*	*B. cereus*	*P. mirabilis*	*P. aeruginosa*	*S. aureus*	*S. tyhpimurium*	*S. marcescens*	*C. albicans*
*B. subtilis*	11.6 ± 0.03	11.3 ± 0.05	10.3 ± 0.05	13.3 ± 0.05	14.0 ± 0.16	11.5 ± 0.13	12.3 ± 0.04	13.3 ± 0.18
*B. coagulans*	12.9 ± 0.07	11.3 ± 0.07	10.4 ± 0.07	9.8 ± 0.12	11.2 ± 0.19	10.6 ± 0.14	13.3 ± 0.05	10.3 ± 0.18
*B. amyloliguefaciens*	13.3 ± 0.03	14.3 ± 0.03	8.8 ± 0.11	13.6 ± 0.14	11.0 ± 0.14	9.5 ± 0.07	11.7 ± 0.07	10.3 ± 0.04
*L. fermentum*	11.6 ± 0.07	12.3 ± 0.03	13.0 ± 0.09	9.1 ± 0.11	10.6 ± 0.13	9.3 ± 0.08	11.3 ± 0.09	9.1 ± 0.19
*L. acidophilus*	8.6 ± 0.14	10.3 ± 0.08	9.3 ± 0.05	10.1 ± 0.11	10.1 ± 0.16	8.9 ± 0.07	11.3 ± 0.08	10.1 ± 0.14

The number of colonies that had grown on the agar medium’s surface served as a measure of the bacterial infestation. Pathogenic microorganisms such as *S. aureus* bacteria were detected in 50% of the samples taken. The bacteria can be spread from cow to cow through contact with contaminated surfaces, such as milking equipment, bedding, and floors [23,24]. Also, in 36% of samples, bacteria of the *E. coli* group, *Enterobacter aerogenes*, and *P. mirabilis* were detected (Table 3).

According to the results, long-term use of the consortium has bactericidal activity against all cultures studied, especially the causative agent of mastitis, *S. aureus*, except *P. mirabilis*, *Proteus vulgaris*, and *E. coli*. As a result, it was found that mainly probiotic culture colonies grew on the agar surface at a dilution of 1:10. But in the 1:100 dilution, colonies of yellow, matte-colored cultures of different sizes were found, but in this case, the growth of probiotic culture colonies prevailed.

In the bodies of animals, bacteria release an antibacterial substance of a protein nature that suppresses the development of pathogenic and conditionally pathogenic bacteria; they produce proteolytic enzymes, an immuno-modulator [25,26].

**Table 2.** Cell titer values before and after centrifugation.

Strain	Initial titer	After centrifugation, rpm		
		4,000	10,000	13,000
*B. subtilis *C	8.5 × 10^8^	9.2 × 10^9^	7.2 × 10^9^	6.5 × 10^9^
*L. acidophilus *B	7.4 × 10^8^	8.7 × 10^9^	7.9 × 10^9^	2.5 × 10^7^

**Table 3.** Contents of bacterial contamination of udder skin and milk wipe (microorganisms in 1 ml of wipe).

Name of isolated microorganisms	Before treatment	After treatment		
		Exposure time		
		48 h	240 h	30 h
*P. aeruginosa*	+	+	+	−
*E. coli*	+	+	+	+
*Streptococcus agalactiae*	+	+	−	−
*Klebsiella pneumonia*	+	+	−	−
*S. aureus*	+	+	−	−
*Streptococcus lactis, Streptococcus pyogenes, Streptococcus cremoris, Streptococcus faecalis, Streptococcus xylosus*	+	−	−	−
*Citrobacter diversus, freundii*	+	−	−	−
*E. aerogenes*	+	+	+	−
*Serratia liquefaciens*	+	−	−	−
*P. mirabilis, P. vulgaris*	+	+	+	+
*Candida*	+	+	−	−

### Effect of probiotic consortium on mastitis prevention 

Mastitis (inflammation of the mammary gland) is the most common disease in dairy cattle. It causes economic losses due to the reduction of milk productivity and the deterioration of milk quality. Inflammation of the udder is caused by incorrect milking and unsatisfactory conditions for housing and feeding cows, which can contribute to the penetration and development of microbes in the mammary gland [27,28]. The most effective way to prevent subclinical mastitis is to implement prophylactic measures such as regular floor cleaning, keeping udders clean, cleanliness of milkmaids, and treatment of cows, especially in high-yielding dairy cows [29,30].

Unlike antibiotics, probiotics are physiologically effective and harmless to animals. The positive effect of probiotics is due to their participation in the processes of digestion and metabolism in the body, biosynthesis, and absorption of proteins and many other biologically active substances, as well as providing resistance to microorganisms [31,32].

The use of probiotic agents leads to an increase in bacterial colonization of the mammary gland teats, while at the same time, there is a predominance of bacteria of the genus *Bacillus* and *Enterococcus* and a decrease in the quantity of opportunistic microflora. In the context of udder health, *Bacillus* and *Enterococcus* may also have beneficial effects, such as reducing the risk of mastitis, a common udder infection in dairy cows. The decrease in opportunistic microflora is a positive outcome, as it suggests that the use of probiotics may help to prevent udder infections and improve udder health overall [33,34].

Probiotics can help support the daily activities of a cow and improve its overall health and performance. Probiotics can support gut health by directly inhibiting potentially harmful bacteria in the gut, which reduces the risk of infections and illnesses. Additionally, probiotics can indirectly support gut health by promoting a healthy gut environment that is less favorable to the growth of harmful bacteria [35,36]. Probiotic culture consortium was used at the following concentrations: 7% for the first week, 5% for the second week, and subsequently applied at a concentration of 3%. The maintenance and housing of cows in both groups were identical. A milking system, the ADM-8, was used for milking. Milking was performed twice a day. For the control group of cows, disinfection of the housing was not performed. The process of udder preparation before milking involved washing it with clean tap water, which was sourced from a shared bucket and applied using a communal washcloth. Following milking, the teats were treated with Dipal, an antiseptic solution containing iodine as an active ingredient, and sorbitol to provide a gentle effect. The preparation was applied to the teats after milking by dipping them into a special cup.

Before the study, among all the teats examined in the experimental group, 85.3% were found to exhibit a physiological response to machine milking. Additionally, hyperkeratosis was detected in 10.8% of the teats, while complicated hyperkeratosis was found in 1.2% of the teats.

After 1 month of administering a consortium of probiotic preparations, the udder teats were reevaluated, and the results revealed a considerable improvement in the condition of the mammary gland tissues, indicating the high efficacy of the probiotic treatment. This is confirmed by a 1.1-fold increase in the number of teats with physiological reactions as well as a 2-fold reduction of such pathological changes as hyperkeratosis. The results of the study of udder teat condition are summarized in Table 4.

The most important indicator of udder treatment consortium efficiency is the level of mastitis prevalence. The studies showed that in the experimental group, there was a decrease in the number of cows with mastitis in one or more quarters by 1.2 times, with a predominantly 1.5 times reduction in the number of cows with clinically pronounced mastitis.

**Figure 4.**
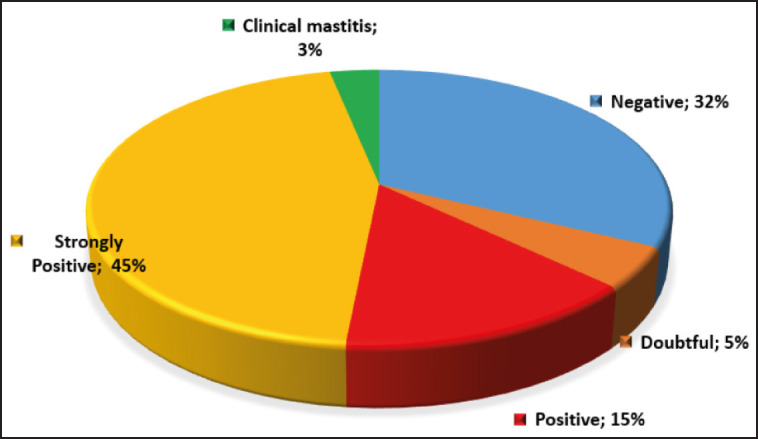
Distribution of mastitis types in cows in Rodina Farm.

As udder teat diseases are highly prevalent, we conducted an investigation into latent mastitis in 41 cows by testing their milk with the Kenotest rapid mastitis test. The obtained results showed a significant prevalence of hidden mastitis in cows in the herd. Thus, positive and strong-positive reactions with the rapid mastitis test were recorded in 14.5% and 45.2% of cows, respectively; negative reactions in 32.2%; and doubtful reactions in 4.8% of cows. Clinical mastitis was observed in two animals, which represented 3.2%. The prevalence of latent mastitis in Rodina AF LLP is presented as a pie chart in Figure 4.

**Table 4.** State of udder teats in cows of Rodina AF LLP.

Stage of experiment	Physiological reaction, %	Mastitis, %
Before	85.3	10.8
After	92.4	5.5

**Figure 5.**
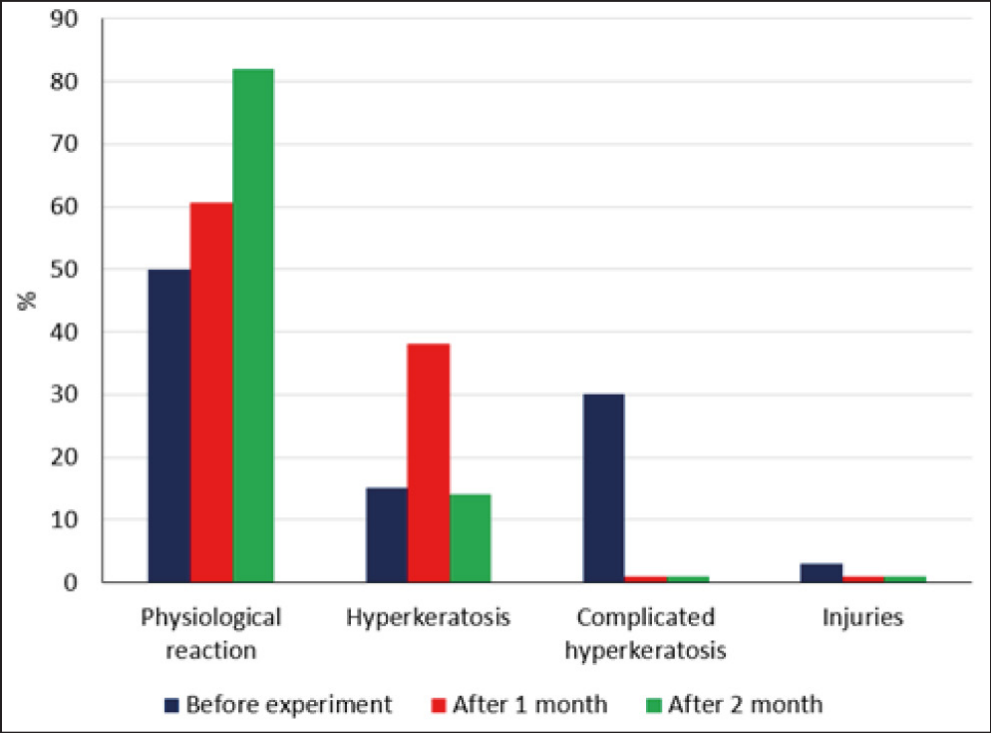
Change of udder teat condition after using probiotic agents.

In order to evaluate the efficacy of probiotics in preventing mastitis, a group of 41 cows was selected and housed together for the experiment. Prior to the study, as well as after 1 and 2 months of administering the prophylactic agents, the presence of latent mastitis in each quarter of the udder was determined using the Kenotest rapid mastitis test. Figure 5 presents the results of the study, indicating a considerable improvement in udder teat condition after 1 month of probiotic treatment. Specifically, 60.7% of teats exhibited a normal physiological reaction to milking, which represents a 1.2-fold increase from the initial values. The occurrence of complicated hyperkeratosis in udder teats decreased to 0.8%, while the number of teats with uncomplicated mastitis increased by 2.4 times as a result of the transition from complicated to uncomplicated forms.

Upon evaluation of the impact of the probiotic consortium on udder health, it was observed that the number of cows exhibiting a sharply positive reaction in udder quarters during the rapid mastitis test decreased to 35.4% with the use of the consortium. Simultaneously, the number of cows exhibiting a negative reaction increased to 9.7%, while those with a doubtful reaction increased to 19.4%. However, there was also an increase in the number of animals with a positive reaction by 1.5 times, and cows exhibiting the clinical form of mastitis increased by two times, from one to two cows.

Table 5 presents the data on the changes in the level of udder inflammatory diseases over time with the use of the probiotic consortium. The results from the study conducted after 2 months of consortium use indicated that the number of cows with a negative or doubtful reaction during the rapid mastitis test remained unchanged. However, due to a decrease in the number of cows with a sharply positive reaction, there was an increase in the number of animals exhibiting a positive reaction from 29% to 41.9%, including those with clinical mastitis.

The analysis of mastitis prevalence by udder parts revealed that after 2 months of probiotic treatment using Kenotest, the number of udder parts exhibiting a positive reaction decreased by 1.4 times. In contrast, the number of quarters showing a negative and doubtful reaction increased by 1.2 times, rising from 51.6% to 62.1%. Furthermore, there was an increase in the number of cases of clinical mastitis, from 1 quarter to 5.

**Figure 6.**
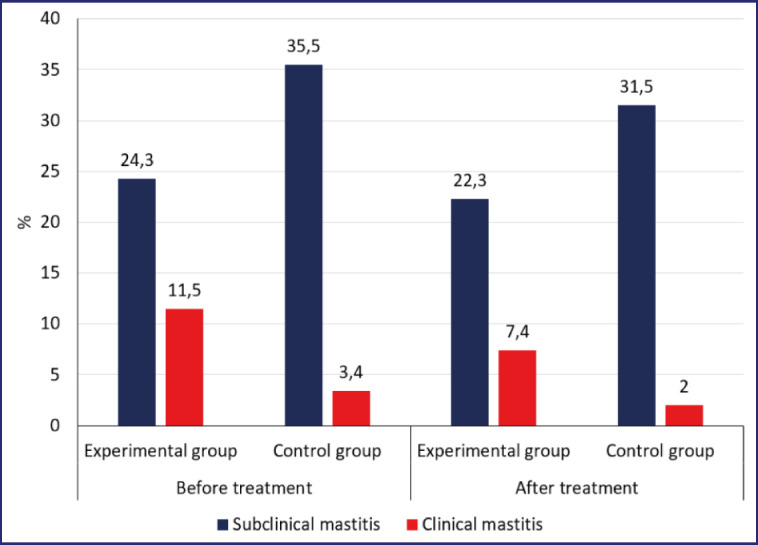
Prevalence of mastitis before and after probiotic treatment.

Analysis of the spread of inflammation by quarters of the udder showed a significant decrease in the number of cases with latent mastitis—by 1.2 times (from 80 to 66) and by 1.6 times the number of cases with clinical mastitis. The prevalence of mastitis following the use of probiotics at “Rodina” Farm is shown in Figure 6.

**Table 5.** Dynamics of change in the level of udder inflammatory diseases when using probiotic agents.

Reaction to the rapid mastitis test	Before experiment		After 1 month		After 2 month	
	Cows	%	Cows	%	Cows	%
Negative	2	6.5	3	9.7	3	9.7
Doubtful	3	9.7	6	19.4	6	19.4
Positive	6	19.4	9	29	13	41.9
Strongly positive	19	61.3	11	35.4	5	16.1
Clinical mastitis	1	3.2	2	6.5	4	12.9

The control group showed a decrease in the number of cows with affected udder quarters. However, when looking at the distribution of mastitis by udder parts, there was no significant change in the number of quarters affected by mastitis during the study period. Before the experiment, there were 75 quarters with latent mastitis and 6 quarters with clinical mastitis, while after the experiment, there were 73 quarters with latent mastitis and 5 quarters with clinical mastitis. The results of Shkromada et al. [37] showed that the use of *B. megaterium* NCH 55 as a probiotic had a positive therapeutic effect on subclinical mastitis in cows. In the study [38], probiotics were used as a therapeutic and prophylactic agent in the early form of mastitis in dairy cows.

### Content of somatic cells in milk samples 

The increased bacterial contamination of milk is the result of not complying with the requirements for the maintenance of cows. Feeding high-quality, balanced feeds and adhering to commonly accepted hygienic requirements for feeding and keeping cattle is the main condition for obtaining high-quality milk. On dairy farms, it is necessary to adhere to certain rules for producing milk with high consumer properties, preventing udder disease, and providing appropriate treatment for sick cows [39,40].

Milk from cows with mastitis can cause infections in humans and animals. Due to the increase of somatic cells, milk becomes less heat-stable, and its technological properties deteriorate [41]. To assess the efficacy of the probiotic consortium, individual milk samples were analyzed from each cow in the experimental and control groups to measure the number of somatic cells present. Milk samples were taken before the experiment and repeatedly after 30 days of using the preparations. Laboratory analysis of samples to determine the level of somatic cells was performed on a DeLaval somatic cell counter, with the fluorescent-optical method of cell determination, every week for 30 days of using the consortium.

**Figure 7.**
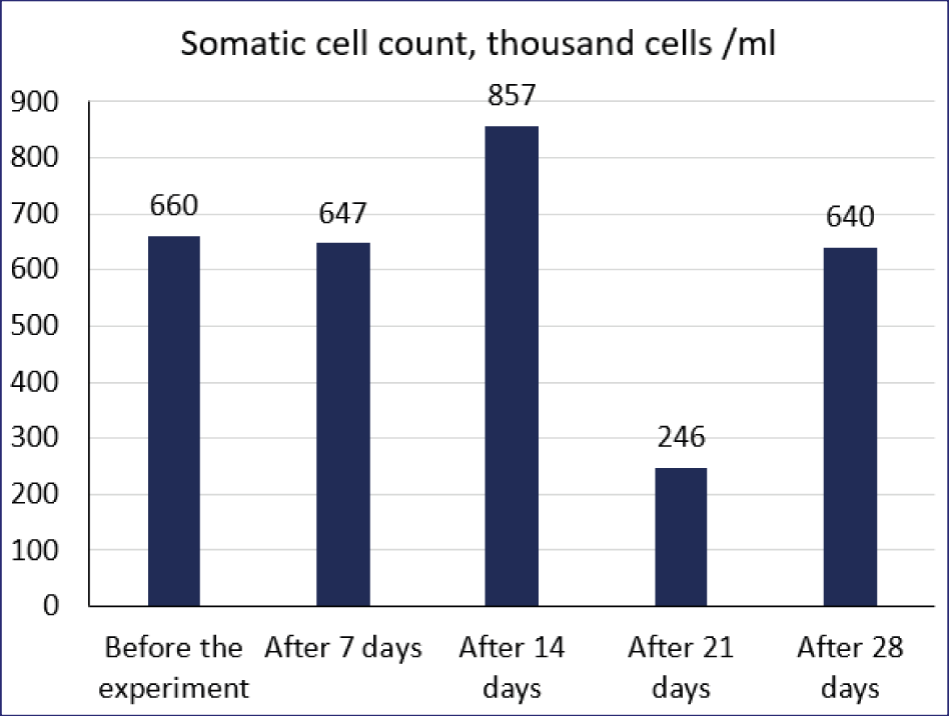
Somatic cell count in milk over the duration of the experiment.

**Table 6.** Indicators of milk yield and somatic cells in experimental and control groups.

Indicator	Before		After	
	Control group *n* = 21	Experimental group *n* = 40	Control group *n* = 21	Experimental group *n* = 40
Milk yield, kg	20.8 ± 0.6	21.2 ± 0.5^a^	19.3 ± 0.6	20.3 ± 0.6
Somatic cells, thousand/ml	264.27 ± 23.31	233.11 ± 19.61^a^	295.98 ± 24.37	280.11 ± 23.78^a^

a The difference is significant compared with a control group, *p* ≤ 0.05.

**Figure 8.**
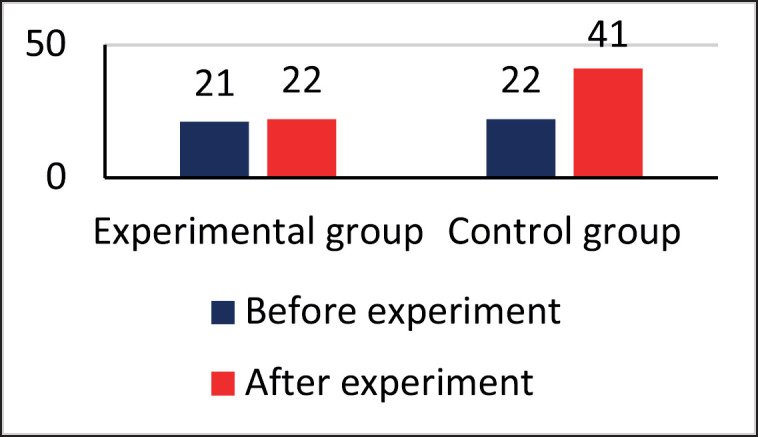
Number of cows with the inflammatory process in one or more udder quarters.

The observed changes in the level of somatic cells in milk were mainly attributed to the violations detected during machine milking. These included the removal of the milking machine without disconnecting from the vacuum, prolonged “idle” milking, an unstable vacuum level that caused marked anxiety in animals, and the presence of blood in the remaining portions of milk, which was determined organoleptically. The level of somatic cells in milk is shown in Figure 7.

The results of the study showed significant fluctuations in the somatic cell count in milk from the experimental group of cows. Prior to the use of the probiotic complex, the somatic cell count was 660,000 cells/ml. There was a sharp increase in the count to 1.3 times the initial level after the application of the complex. After 14 days of using the complex, the somatic cell count decreased by 3.2 times to 246,000 cells/ml. However, the count increased again to the initial level of 640,000 cells/ml.

A slight reduction in milk quantity was observed in both the experimental and control groups. Additionally, there was an increase in somatic cell count of 1.2 times in the experimental group and 1.1 times in the control group. A summary of the data analysis can be found in Table 6.

**Figure 9.**
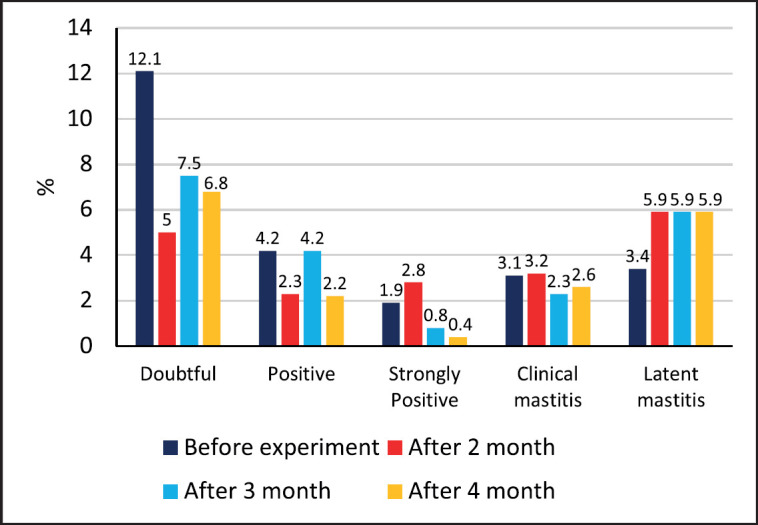
Prevalence of different forms of mastitis when using probiotic consortium.

In healthy cows, the physiological norm of somatic cell content is between 100 and 170 thousand/cm^3^. Often, this number depends on the individual characteristics of the animal [42,43]. Additionally, we analyzed the results with a somatic cell count of more than 1,000 thousand cells/ml, which is evidence of the development of the inflammatory process in one or several quarters of the mammary glands.

The data indicated that there was no notable difference in the number of cows with high levels of somatic cells in the experimental group before (21 cows) and after (22 cows) the probiotic consortium was administered (Fig. 8). On the other hand, in the control group, there was a significant increase in diseased animals, from 11 cows before the experiment to 22 cows after 1 month. The distribution of latent mastitis by quarters of the udder when using the consortium is shown in Figure 9.

Based on the research conducted, it can be concluded that administering probiotic microorganisms as an active ingredient has a significant positive impact on the udder tissue of lactating cows. The effectiveness of the treatment protocol, which includes sanitation of the livestock building, feed, and water, as well as direct udder treatment before and after milking using the consortium, was observed. The use of a consortium containing probiotic microorganisms has a more rapid positive effect on the mammary gland than when only using agents for mastitis prevention. However, using only mastitis prevention agents can still lead to a reduction of latent mastitis and udder teat hyperkeratosis in the herd, but this is mainly achieved with prolonged use of the agents for at least 3–4 months.

## Conclusion

The technology for obtaining biomass to create a consortium has been developed. It is recommended to use the complex bacterial preparation as a sanitizing agent for preventive measures. The results of the studies allow us to recommend veterinary specialists for agricultural facilities in the organization of preventive measures. The obtained results of the research can be recommended for the disinfection of dairy farm premises in order to prevent mastitis and improve the sanitary and hygienic indicators of milk. The developed technology of creating a consortium of probiotic cultures can be offered to production as an effective tool for the sanitation of livestock facilities. We have proposed a biological agent using probiotic cultures to be used as a cleaning agent for livestock facilities, as well as for the prophylactic treatment and prevention of mammary gland diseases like mastitis in highly productive cows.

## List of abbreviations

DNA, deoxyribonucleic acid; LLP, Limited Liability Partnership; MRS Man, Rogosa, and Sharpe agar; MPA MacConkey plate agar; PCR, polymerase chain reaction; PIP AHS, Pip animal housing stabilizer; PIP CTC, Pip cow teat cleaner; RPM, revolutions per minute.
